# Massive Delayed Vaginal Hemorrhage after Laparoscopic Supracervical Hysterectomy

**DOI:** 10.1155/2012/871041

**Published:** 2012-08-07

**Authors:** M. Brigid Holloran-Schwartz, Shannon J. Potter, Ming-Shian Kao

**Affiliations:** ^1^Department of Obstetrics, Gynecology and Women's Health, Saint Louis University School of Medicine, 6420 Clayton Road, Ste 290, St. Louis, MO 63117, USA; ^2^Division of Gynecology Oncology, Department of Obstetrics, Gynecology and Women's Health, Saint Louis University, School of Medicine, 6420 Clayton Road, Ste 290, St. Louis, MO 63117, USA

## Abstract

*Background*. A known complication of supracervical hysterectomy is cyclical bleeding from the retained cervix when functioning endometrial tissue is not totally removed. We present a rare case of delayed postoperative vaginal hemorrhage after supracervical hysterectomy. *Case*. A 44-year-old woman presented on postoperative day 15 after laparoscopic supracervical hysterectomy with massive vaginal hemorrhage requiring emergent re-operation. Her bleeding was controlled with vaginally placed sutures. Ultrasound confirmed no intraperitoneal free fluid. The etiology was thought to be induced by postoperative tissue necrosis from cautery applied to the endocervical canal during the original surgery. *Conclusion*. Delayed vaginal hemorrhage from a retained cervix is a rare complication of laparoscopic supracervical hysterectomy. Caution should be exercised when cauterizing the endocervical canal as induced tissue necrosis may increase the risk of postoperative bleeding.

## 1. Introduction

One of the complications of supracervical hysterectomy is continued cyclical bleeding from the cervical stump in 0–25% of cases [1]. To decrease this risk, some authors recommend routine coagulation of the endocervical canal [1–7]. Cases of intraperitoneal bleeding requiring reoperation have been described, as well as delayed vaginal bleeding from the vaginal cuff as late as 13 days after total laparoscopic hysterectomy (TLH) [2, 8]. Although one study described a case of vaginal bleeding from colpotomy incision 6 days after laparoscopic supracervical hysterectomy [5], to our knowledge, there has not been a report of massive delayed postoperative hemorrhage confined to the vagina after supracervical hysterectomy which required reoperation.

## 2. Case Presentation

M. L. is a 44-year-old G0 Caucasian woman who presented for laparoscopic supracervical hysterectomy (LSH) for large fibroid uterus after counseling on all options including myomectomy and total laparoscopic hysterectomy.

LSH of 1570 gm uterus was performed without difficulty with estimated blood loss of 100 cc. Bipolar cautery was applied to a depth of 5 mm in the remaining endocervix. She was discharged home the morning of postoperative day (POD) 1.

Routine visit on POD 8 was unremarkable, and the patient had no complaints. She called on POD 14 after noting a self-limited episode of vaginal bleeding and a vaginal odor. Evaluation in the office on POD 15 revealed scant brown discharge. The cervix appeared closed and normal except for a 2 mm area of cautery effect at the 3-o'clock site in the transformation zone. There was no cervical motion tenderness. Clue cells were noted on wet mount, and she was started on metronidazole.

The patient called later that same evening with a slow continuous flow of vaginal bleeding. Upon arrival to the emergency room (ER), she began having heavy vaginal bleeding. Evaluation revealed the cervix 1 cm dilated with brisk bright red bleeding. The top of the internal os was noted to be closed after gentle palpation with a Q-tip. The bleeding was thought to be coming from the left side of the endocervix. She was without any other complaints and had a completely benign abdominal exam. After 30 min of unsuccessful attempts in the ER to stabilize the bleeding with pressure, Monsel's solution, silver nitrate and suture, EBL was 1000 cc, and she was taken to the operating room for exam under anesthesia (EUA) and possible exploration.

She had just eaten a full meal prior to arrival, had no abdominal tenderness, and was not felt to be bleeding intraperitoneally. She was given IV sedation for EUA. Deep figure eight sutures were placed vaginally in the cervix at the lateral aspects of the internal os. A running locking stitch was placed around the circumference of the open endocervix. Excellent hemostasis was noted. Intraoperative ultrasound revealed no intraperitoneal free fluid. The vagina was not packed, and the patient was observed overnight.

 Minimal spotting was noted overnight, and she was discharged on POD 1. Follow-up exam 4 days later revealed a slow, persistent, dark flow emerging from the endocervix. She kept a bleeding diary and ultimately stopped bleeding 7 days after the second surgery. She had normal clotting studies. Her recovery thereafter was uneventful.

## 3. Discussion

This case is an unusual presentation of massive postoperative endocervical hemorrhage, limited to the vagina, without intraperitoneal bleeding. It is possible that the tissue necrosis induced by bipolar cautery of the endocervix resulted in a situation similar to delayed bleeding from a cone biopsy. Most cases of delayed hemorrhage after cold knife cone occurred 10–12 days postoperatively in one study [9], although it occurred as late as postoperative day 31 according to a study by Larsson et al. [10]. The descending branches of the uterine artery supply blood to the cervix and are typically located laterally. Sutures, if used, can be placed laterally during a cold knife conization to minimize blood loss.

An inciting event is difficult to ascertain. Intercourse was denied in both our case and others in the literature [5]. Postoperative bleeding is not reported as a complication of bacterial vaginosis (BV) [11], although one might speculate this as a contributing factor. The prevalence of cyclical bleeding from the cervical stump occurs in up to 25% of patients. The higher rates have been attributed to amputation above the cervical os [1]. In our case, endocervical glands were present on pathology, indicating that amputation occurred below the internal os and below the insertion of the uterine artery. Various techniques have been described to prevent postoperative LSH cyclical spotting. Cauterization of the endocervix has been well described in the literature [1–7]. Coring of the endocervical canal has been recommended by some [1, 7]; conversely, the excessive use of cautery has been proposed as a risk factor for vaginal vault problems [2, 12].

 In our current case, bipolar cautery was applied to a depth of 5 mm in the remaining 3.5 cm endocervix for approximately 10 seconds. A 2 mm area of cautery effect at the 3-o'clock site in the transformation zone was noted on POD 15, and it appeared that bleeding was coming from the left side of the endocervix on exam in the ER. Thus, it seems reasonable to recommend that caution should be taken when applying energy into the endocervix for the potential lateral thermal spread and tissue necrosis. These can be contributing factors for delayed postoperative bleeding.
